# Molecular determinants for Rous sarcoma virus intasome assemblies involved in retroviral integration

**DOI:** 10.1016/j.jbc.2023.104730

**Published:** 2023-04-20

**Authors:** Sibes Bera, Ke Shi, Hideki Aihara, Duane P. Grandgenett, Krishan K. Pandey

**Affiliations:** 1 Department of Molecular Microbiology and Immunology, School of Medicine, Saint Louis University, St Louis, Missouri, USA; 2 Department of Biochemistry, Molecular Biology and Biophysics, University of Minnesota, Minneapolis, Minnesota, USA

**Keywords:** retrovirus, Rous sarcoma virus, integrase, cryo-electron microscopy, DNA-protein interaction, structure-function, intasome structure, HIV-1 strand transfer inhibitors

## Abstract

Integration of retroviral DNA into the host genome involves the formation of integrase (IN)–DNA complexes termed intasomes. Further characterization of these complexes is needed to understand their assembly process. Here, we report the single-particle cryo-EM structure of the Rous sarcoma virus (RSV) strand transfer complex (STC) intasome produced with IN and a preassembled viral/target DNA substrate at 3.36 Å resolution. The conserved intasome core region consisting of IN subunits contributing active sites interacting with viral/target DNA has a resolution of 3 Å. Our structure demonstrated the flexibility of the distal IN subunits relative to the IN subunits in the conserved intasome core, similar to results previously shown with the RSV octameric cleaved synaptic complex intasome produced with IN and viral DNA only. An extensive analysis of higher resolution STC structure helped in the identification of nucleoprotein interactions important for intasome assembly. Using structure-function studies, we determined the mechanisms of several IN–DNA interactions critical for assembly of both RSV intasomes. We determined the role of IN residues R244, Y246, and S124 in cleaved synaptic complex and STC intasome assemblies and their catalytic activities, demonstrating differential effects. Taken together, these studies advance our understanding of different RSV intasome structures and molecular determinants involved in their assembly.

Integration of retroviral DNA into the host genome, an essential step in retroviral replication, is mediated by the viral integrase (IN). Upon retrovirus infection, the RNA genome is converted to dsDNA by the viral reverse-transcriptase (RT). Both the IN and RT are closely associated with the viral genome within the virion core particle. IN binds to both ends of the linear viral DNA and removes two nucleotides from the 3′-ends adjacent to conserved CA dinucleotides exposing the reactive 3′-OH group. In the integration reaction, termed strand transfer, IN joins the processed viral 3′-OH ends with a scissile phosphate on the target DNA strands through a S_N_2-type nucleophilic substitution. This joining to the host DNA occurs in a staggered manner separated by four to six bp, a unique characteristic of different retroviral species (1).

IN from Rous sarcoma virus (RSV), HIV-1, and related retroviruses share a three-domain organization, namely a catalytic core domain (CCD) flanked by amino-terminal domain (NTD) and carboxy-terminal domains (CTD) (2). The NTD folds into a three-helix bundle and contains the conserved HHCC motif which coordinates a Zn^2+^ ion. The CCD is the most conserved domain among retroviruses and contains the active site comprising the DDE (Asp-Asp-Glu) motif which is responsible for catalytic activity. The CTD adopts an SH3 fold and is the least conserved domain. This domain may be the primary factor for producing different oligomeric forms of nucleoprotein complexes observed with different retroviral INs. The C-terminal tail region which extends beyond the CTD is flexible and has not been resolved in any IN structure. The tail region (17 aa) in RSV IN appears to have a major role in forming specific IN–DNA complexes termed intasomes. Truncations of the C-terminal tail of RSV IN favors the accumulation of a precursor tetrameric intasome en route to the mature octameric cleaved synaptic complex (CSC) intasome (3, 4, 5).

Purified retroviral IN exists in different oligomeric forms, ranging from monomers to dimers to tetramers and higher order species. Virion-derived or recombinant RSV IN is predominantly dimeric (6), while recombinant HIV-1 IN is either monomeric, tetrameric, or a mixture thereof (7, 8, 9, 10, 11). The oligomeric forms of free IN or IN associated with viral RNA or DNA *in vivo* remains unknown. IN multimerizes onto viral DNA ends to produce a series of complexes in the concerted integration pathway *in vitro* (12).

IN binds to both ends of linear viral DNA (produced upon reverse-transcription) and in a reaction termed 3′-processing, removes two nucleotides from the 3′-ends producing CSC (Fig. 1*A*). IN within the CSC binds host DNA to form the strand transfer complex (STC) and mediates the nicking of host DNA and joining of viral DNA to host DNA. Collectively, the CSC, STC and their intermediate complexes are termed intasomes. In general, most intasome assemblies observed *in vitro* suggest DNA-mediated tetramerization of the predominant IN species observed in solution. Monomeric prototype foamy virus (PFV) IN assembles tetrameric intasomes (13), while dimeric α- and β-retroviral INs form octameric intasomes (14, 15). Lentiviral INs like HIV-1, Maedi-visna virus, and simian immunodeficiency virus are mostly tetrameric and have been observed or expected to produce hexadecameric intasomes (16, 17, 18, 19).

**Figure 1:**
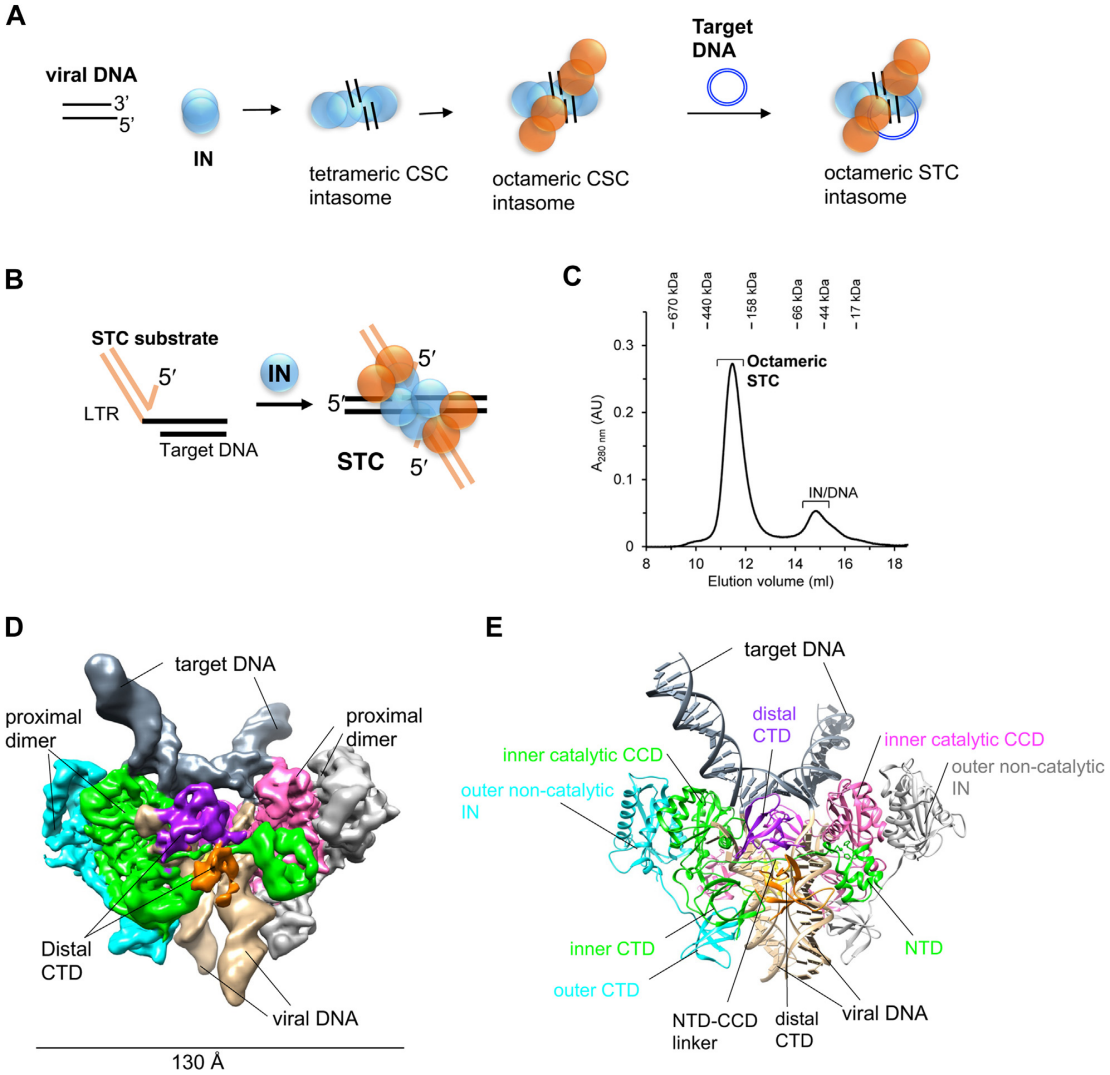
**Cryo-EM structure of the RSV STC.***A*, schematics of RSV intasome assembly. The WT full-length IN (1–286 aa) binds to viral LTR DNA ends, resulting in the assembly of a transient intermediate-tetrameric CSC intasome. IN is shown as dimer. Binding of additional IN molecules in distal positions results in the formation of a stable octameric CSC. In presence of a target DNA, an octameric strand transfer complex (STC) is formed. Proximal and distal IN protomers are shown in *blue* and *orange*, respectively. *B*, schematics of the STC substrate and RSV IN to assemble STC intasomes for cryo-EM. *C*, a typical size-exclusion chromatography (SEC) profile of STC purification. The elution positions of molecular weight standards are marked. *D*, cryo-EM density of the RSV STC. Each of eight IN subunits is colored differently. Target and viral DNAs are marked and shown in *slate gray* and *tan color*, respectively. *E*, model of the STC structure using same colors as in D. The proximal chains A, B, E, and F are shown in *pink*, *gray*, *green*, and *cyan*, respectively. The distal chains C, D, G, and H are shown in *purple*, *orange*, *magenta*, and *yellow*, respectively. CSC, cleaved synaptic complex; IN, integrase; LTR, long terminal repeat; RSV, Rous sarcoma virus.

Intasome assembly mechanisms are not clearly defined. We used RSV IN as a model system to determine its intasome structures and to understand their assemblies and associated functions. We hypothesize there are key intermediates in the intasome assembly pathway with IN multimers and viral DNA that subsequently binds target DNA resulting in integration. Our previous studies demonstrated that an RSV tetrameric CSC intasome is the precursor to mature octameric CSC intasome (4, 5). The ability of RSV IN to assemble the catalytically active octameric intasome from its tetrameric precursor is unique among studied retroviral systems.

Previously, we determined the structure of RSV octameric CSC intasome by cryo-EM (20). The four proximal IN subunits complexed to the viral DNA ends form the conserved intasome core (CIC) (12), while the four distal subunits showed dynamic flexibility. We hypothesized that this flexibility could be stabilized by target DNA binding in the STC intasome, as shown in its X-ray crystal structure determined at 3.86 Å resolution (15). Cryo-EM captures DNA–protein complexes in their more native dynamic form, while X-ray crystallography captures these complexes in a single more rigid conformation assisted by the crystal lattice. To resolve any potential structural bias, we determined the structure of RSV STC intasome in its native state by cryo-EM at an improved overall resolution of 3.36 Å. We carried out site-directed mutagenesis of selected IN residues to determine their effect on different intasome assemblies and catalytic functions. Collectively, these data advance the structural understanding of different RSV intasomes and molecular determinants involved in their assemblies.

## Results

### Cryo-EM structure of the RSV STC

STC intasomes were assembled using a DNA substrate that contains viral DNA covalently joined to target DNA (Fig. 1*B*). Upon integration, RSV produces a 6 bp host-site duplications at both ends of the cellular integration site. We used a similar 6 bp cellular sequence covalently linked at the 3′-end to the conserved CA residue at the viral long terminal repeat (LTR) ends, thus mimicking the integration product. STC intasomes were purified by size-exclusion chromatography (SEC) using a Superdex 200 Increase column (10 × 300 mm) (Fig. 1*C*). Unlike most other retroviral intasomes, we did not observe higher order species of STCs. RSV IN produced predominantly homogeneous octameric STCs (Fig. 1*C*). Similarly, homogeneous mature octameric CSC intasome is assembled with RSV IN and viral DNA (20). Top fractions of STCs from the SEC column were used for vitrification and cryo-EM imaging on Titan Krios microscope using Falcon 4 camera (Table S1). We determined the structure of RSV STC by single particle cryo-EM (Figs. 1*D* and S1 and Table S1). We used cryoSPARC (21) to perform iterative 2D and 3D classification on our dataset of 3297 movies. We subsequently obtained a 3D map at 3.36 Å resolution from a stack of 141,428 particles (Fig. 1*D*). The CIC region that contained the viral and target DNA interactions within IN active sites had a resolution of ∼ 3 Å (Fig. S1*H*). The cryo-EM structure demonstrated the presence of four IN dimers bound to the two molecules of STC DNA substrates, modeled in Figure 1*E*. The proximal subunits which have extensive interactions with DNA and the CTD region of distal subunits both had clear density and were well resolved. However, the distal NTD-CCD regions were resolved at a lower resolution probably due to dynamic flexibility observed also in this region with RSV CSC intasomes (20).

### Interactions between RSV IN and DNA in the STC intasome

IN binding of the target DNA in the CSC intasome stabilizes this complex and produces the STC. We used a computational program DNAproDB (22) to carry out an extensive *in-silico* analysis to identify the potential IN residues that have interactions with DNA in the STC. Most of these interactions were with viral DNA which included contacts with either the nucleotide base only including the major and minor groove (Fig. S2) or sugar-phosphate backbone (Fig. S3). The majority of the IN interactions were limited to the terminal 10 nucleotides of the nontransferred viral DNA strand.

**Figure 2:**
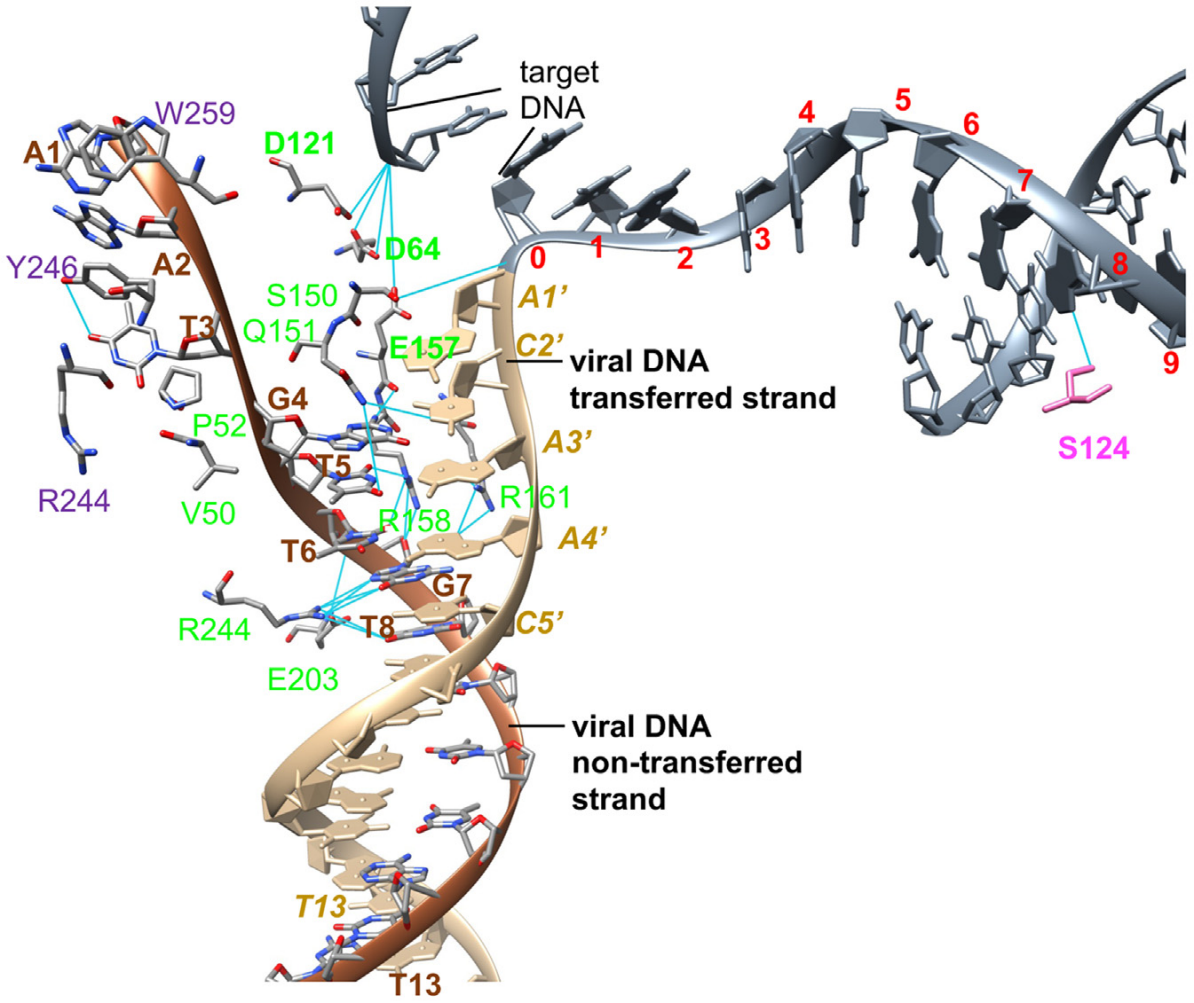
**Visualization of selected interactions between IN and DNA in the RSV STC**. The two viral DNA strands are depicted in distinct colors. The target DNA (shown in *slate gray* color) is covalently joined to the transferred strand of viral DNA. The select IN residues of proximal and distal subunits interacting with viral and target DNA nucleotides are colored by their respective protein chain as in Figure 1*E*. The catalytic triad of active sites D64, D121, and E157 is indicated as *bold dark green* residues. IN, integrase; RSV, Rous sarcoma virus; STC, strand transfer complex.

The NTD-CCD linker of the inner proximal subunit swaps across the CIC in the STC intasome and interacts with viral DNA bound to another unit of proximal dimer in the CIC (Fig. 1, *D* and *E*). The linker residues V50 and P52 interact with T3 and G4 of the nontransferred DNA strand (Fig. 2). Not surprisingly, the proximal inner subunits (shown in green) which provide the active site for catalysis had maximal interactions with the viral DNA nucleotides. At the same time, the outer proximal subunit and distal subunits of IN also had interactions with the viral DNA. There were multiple viral DNA contacts with the distal CTD subunits. For example, the W259, R244, and Y246 (shown in purple) interact with the 5′-terminal nucleotides on the nontransferred DNA strand (Figs. 2 and S4).

Compared to several interactions between IN and viral DNA, there were minimal nucleotide base–specific contacts in target DNA (Figs. 2 and S2). Importantly, S124 of the inner proximal subunits interact with a nucleotide at the eighth position from the cleavage site (Fig. S4). However, there were several interactions between IN residues and sugar phosphate backbone of the target DNA, albeit in region beyond the six nucleotides after the cleavage site (Fig. S3). The six nucleotides immediately adjacent to the conserved CA cleavage site had no interactions with IN suggesting the flexibility to accommodate near random integration sites (23).

### Biochemical characterization of IN residues interacting with viral DNA

**Figure 3:**
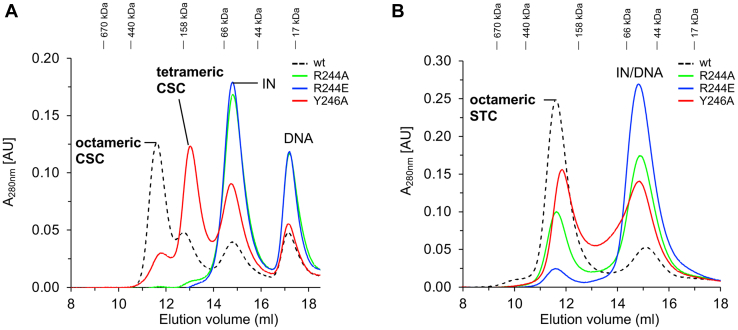
**Effects of substitutions at select IN residues interacting with viral DNA for CSC and STC intasome assemblies**. *A*, MK-2048–trapped CSC intasomes were assembled with WT RSV IN or its mutants R244A, R244E, and Y246A at 18 °C for 18 h. The assembled CSC intasomes were analyzed by SEC. *B*, the STC intasomes were assembled for 18 h at 4 °C with the above IN proteins and analyzed by SEC. Elution positions of molecular weight markers are indicated. AU, arbitrary units; CSC, cleaved synaptic complex; IN, integrase; RSV, Rous sarcoma virus; SEC, size-exclusion chromatography; STC, strand transfer complex.

**Figure 4:**
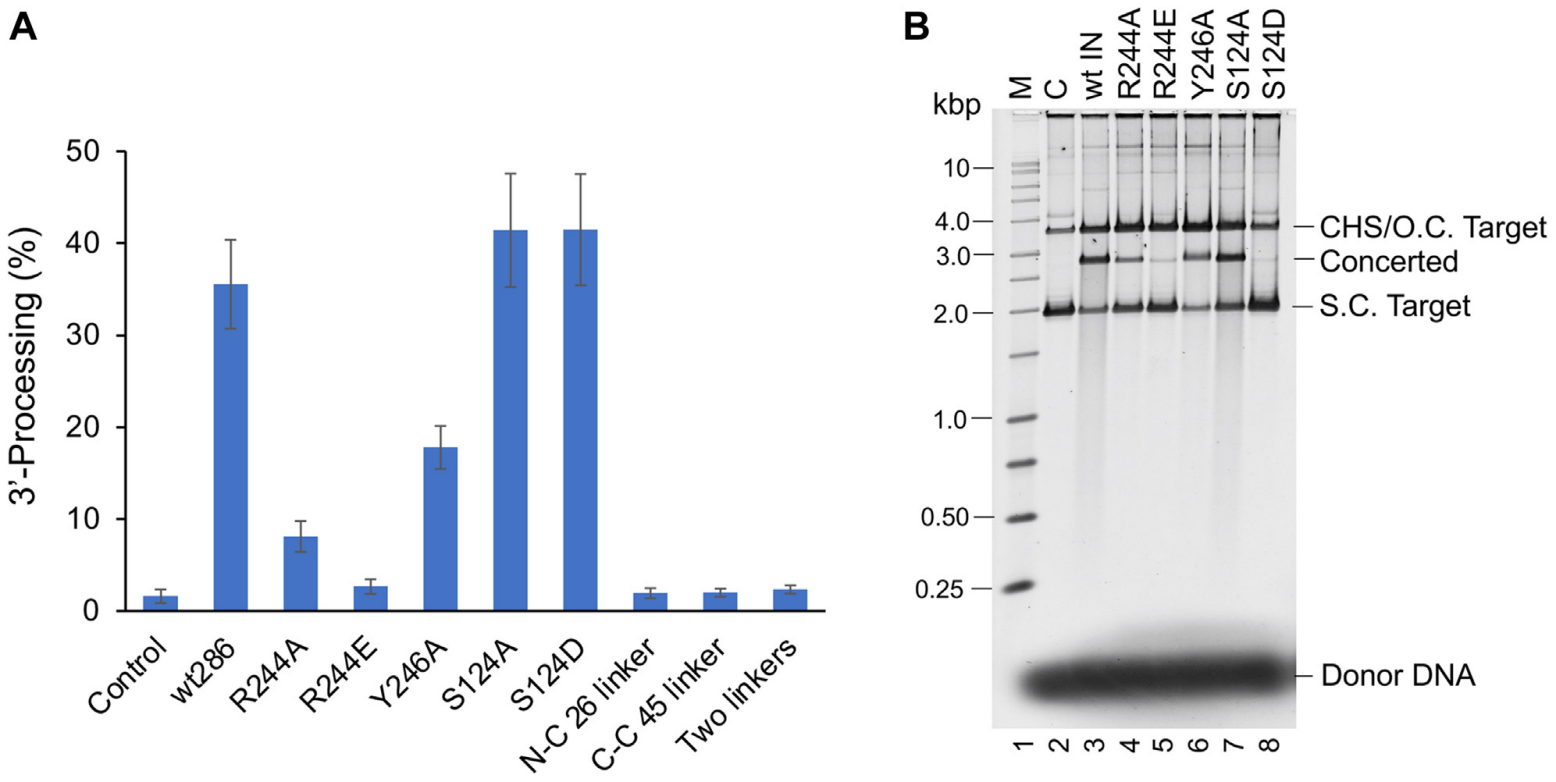
**Catalytic activities of RSV WT and mutant IN**. *A*, 3′-processing activities of WT IN (1–286) and designated mutant IN proteins were determined using 20 nM IN and 0.5 nM of ^32^P-labeled blunt-ended DNA substrates at 37 °C for 30 min. The SD was determined from at least three independent experiments. *B*, the concerted activity of each IN mutant was determined with 18R GU3 substrate at 37 °C for 30 min. The products were deproteinized and run on a 1.3% agarose gel. Lane 1, marked M contains the molecular size markers (Promega kb ladder). Lane 2, marked C does not contain IN. lanes 3 to 9 contain different IN as indicated on the top. Unused donor DNA is indicated at *bottom right*. CHS, circular half-site; IN, integrase; RSV, Rous sarcoma virus; s.c. supercoiled; o.c. open circular.

Based on the cryo-EM structures of the RSV STC (Fig. 1) and CSC intasome (20), we selected IN residues for site-directed mutagenesis to determine their role in intasome assembly (Fig. 3) and catalytic activities (Fig. 4). We expressed and purified RSV IN mutants R244A, R244E, and Y246A that interact with the 5′-terminal viral DNA nucleotides of the nontransferred strand (Figs. 2 and S4). Similar to WT IN (1–286), all of these IN mutants were dimeric (Fig. S5). We determined the ability of these IN mutants to interact with viral DNA to produce CSC and STC intasomes (Fig. 3). We used longer assembly times and conditions that allow the intermediate tetrameric CSC intasome to be effectively converted into the octameric form by WT IN (Fig. 3*A*) (4,5). Under these conditions, IN mutants R244A and R244E were completely defective in producing tetrameric and octameric CSC intasomes, while IN Y246A produced primarily tetrameric CSC intasomes (Fig. 3*A*). These results suggest that the recruitment of distal dimers to assemble octameric CSC intasomes is compromised with R244A, R244E, and Y246A but allow the latter to produce the tetrameric CSC. IN mutant R244E was unable to assemble STC, while R244A and Y246A were diminished in their ability to produce STC compared to the WT IN (1–286) (Fig. 3*B*). These intasome assembly results matched with the anticipated catalytic activities of these IN mutants. The catalytic activities were determined by measuring the 3′-processing and strand transfer activities to produce concerted integration products (Fig. 4 and Table 1). Using blunt-ended viral DNA substrates, the 3′-processing assays were performed in the presence of Mg^++^ as the divalent metal ion needed for catalysis. The WT IN (1–286) showed ∼35% of 3′-processing activity (Fig. 4*A* and Table 1). The mutant R244A had reduced 3′-processing activity using the blunt-ended substrate (Fig. 4*A*) as well as reduced concerted integration activity determined with a 3′-OH recessed 18R substrate (Fig. 4*B*). R244E was devoid of 3′-processing and concerted integration activities. Y246A was partially active (∼50% of WT IN) for 3′-processing (Fig. 4*A*) and for concerted integration using the 3′-OH recessed substrate (Fig. 4*B*). As mentioned, Y246A is also defective in assembly of octameric CSC intasome, suggesting it is deficient in recruiting the distal subunits. All of these IN mutants were defective to various degrees for assembling the STC intasome, suggesting multiple functions associated with these residues, in contrast to other IN single-point mutants that do not affect assembly of the STC intasome (5).

**Table 1:** Biochemical characteristics of IN residues described in this study

RSV IN	3′-processing	Concerted integration	CSC assembly	STC assembly
wt IN 1–286	++++	++++	++++	++++
R244A	++	++	−	++
R244E	−	−	−	−
Y246A	++	++	+	+++
S124A	++++	++++	++++	++++
S124D	++++	−	++++	−
N-C (26 aa)	−	−	−	−
C-C (45 aa)	−	−	−	−
NC/CC (26/45 aa)	−	−	−	−

The experimental details for 3′-processing using a blunt-ended substrate, concerted integration activity, CSC and STC assembly with a recessed 18R substrate are described in the text. The 3′-processing and concerted integration activities and yield of CSC and STC intasome is presented here relative to the WT IN (1–286 IN).

“++++”, 75 to 100%; “+++, 50 to 75%; “++”, 25 to 50%; “+”, 10 to 25 %; “-”, less than 10%.

### Interactions of RSV IN residues with target DNA

**Figure 5:**
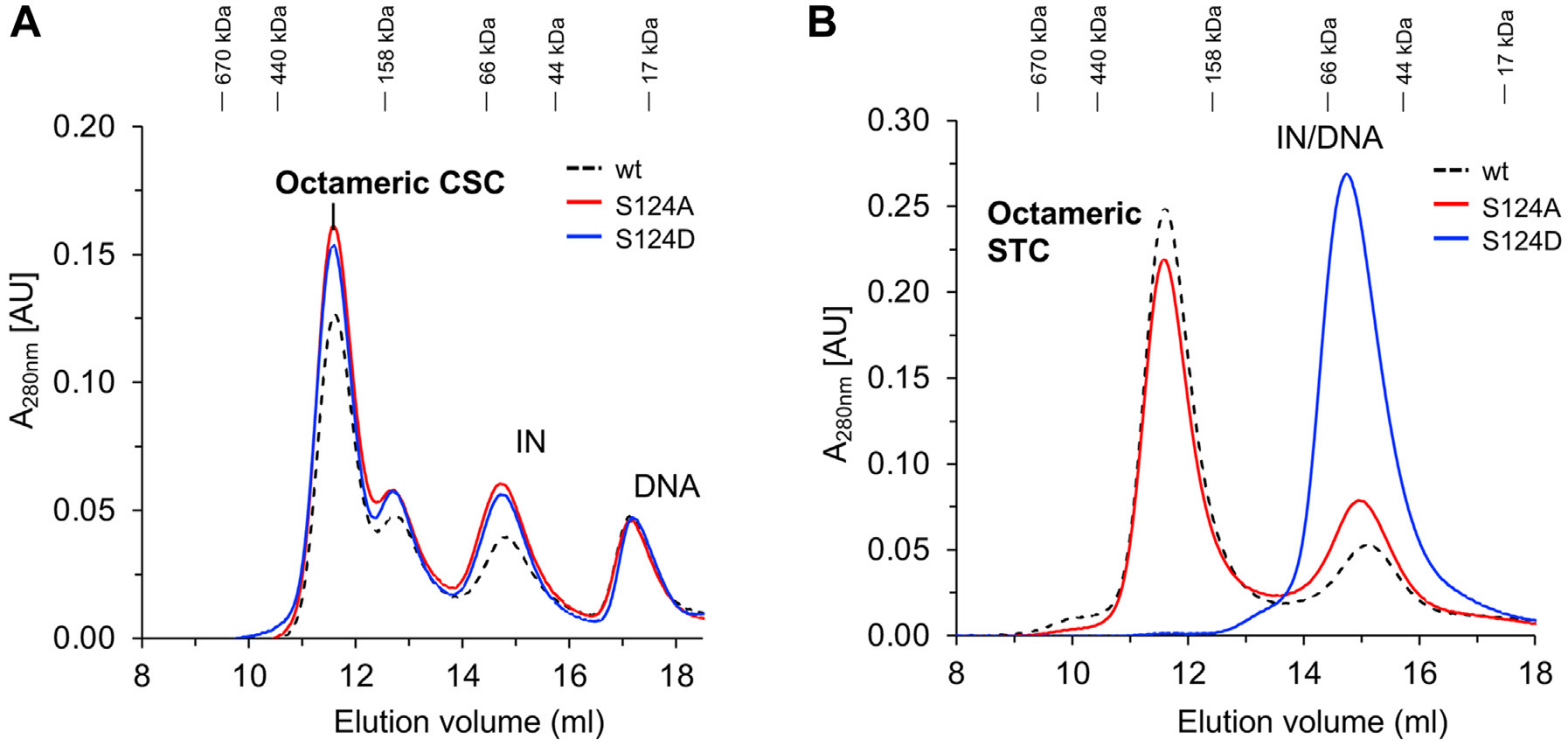
**Analysis of S124 substitutions on CSC and STC intasome assemblies**. *A*, MK-2048–trapped CSC intasomes were assembled with WT RSV IN (1–286) and IN mutants S124A or S124D at 18 °C for 18 h. The assembled CSC intasomes were analyzed by SEC. *B*, the STC intasomes were assembled for 18 h at 4 °C with these same IN proteins and analyzed by SEC. Elution positions of molecular weight markers are indicated. AU, arbitrary units; CSC, cleaved synaptic complex; IN, integrase; RSV, Rous sarcoma virus; SEC, size-exclusion chromatography; STC, strand transfer complex.

We observed minimal nucleotide specific interactions between IN and target DNA (Figs. 1*E* and S2–S4). Previous data suggested that RSV IN S124 played a direct role in target DNA binding (24). We determined that S124 of the inner catalytic subunit interacts in the minor groove with the third nucleotide located six nucleotides from the host duplication sequence. In addition, S124 has several interactions with the target DNA backbone sugar and phosphate groups. We made substitutions at S124 to a neutral amino acid alanine or to a negatively charged residue aspartic acid. As expected, these substitutions did not affect CSC intasome assembly since S124 interactions were presumably limited to target DNA only (Fig. 5*A*). IN mutant S124A showed similar efficiency to assemble STC, while S124D was completely deficient in producing the STC (Fig. 5*B*). Correspondingly, the IN mutant S124A had similar 3′-processing and concerted integration activity comparable to WT IN (Fig. 4). In sharp contrast, S124D had 3′-processing activity similar to WT IN (Fig. 4*A*) but was devoid of concerted integration activity (Fig. 4*B*). The results demonstrated that S124 is important for binding to target DNA and assemble the octameric STC.

### Role of interdomain linker regions in RSV intasome assembly

It has been speculated that the length of the interdomain linker may determine the oligomeric form of retroviral intasomes. PFV IN has relatively longer interdomain linkers compared to RSV, mouse mammary tumor virus (MMTV), and HIV-1. To determine the role of linker length in RSV IN, we modified the NTD-CCD as well as CCD-CTD linker lengths (13 and 8 aa, respectively) in RSV IN to mimic PFV IN either individually or together. RSV IN (1–286) constructs possessing a 26 aa NTD-CCD linker (N-C 26) or a 45 aa CCD-CTD linker (C-C 45) or both of these linkers (NC/CC) were constructed. All linker-modified INs were dimeric (Fig. S5). Surprisingly, all three linker-modified IN constructs were inactive for 3′-processing (Fig. 4*A*) as well as concerted integration (data not shown). The alterations in interdomain linker lengths also abolished the CSC and STC intasome assemblies. These data suggest that interdomain linkers in different retroviral INs have evolved naturally with the requirement of possessing specific linker sizes for their intasome assembly and catalytic activities.

## Discussion

In the RSV concerted integration pathway, IN binds to the viral DNA ends producing the intermediate tetrameric CSC intasome followed by the formation of the mature octameric CSC. The mature octameric CSC binds target DNA followed by strand transfer producing the STC intasome. We assembled the STC intasome that contains IN, bound with a covalently linked viral/target DNA substrate. In this study, we determined the structure of RSV STC intasome by single-particle cryo-EM and investigated structure-function relationships between the CSC and STC intasomes. We previously determined the structure of octameric CSC stabilized by the INSTI MK-2048 by single particle cryo-EM (20). Three-dimensional variability analysis of the RSV STC structure demonstrated significant flexibility of the distal subunits similar to the RSV octameric CSC intasome produced with IN and viral DNA only (20). The conformational flexibility of distal subunits specifically in the NTD-CCD regions in the STC was responsible for insufficient electron density coverage (Fig. S1*D*). We speculated that this flexibility of distal subunits is required to accommodate random target DNA sequences for integration.

We previously determined the structure of RSV octameric STC by X-ray crystallography (15). The crystal structure contained IN (1–270), which possessed a multitude of single-point substitutions C23S, L112M, L135M, L162M, L163M, L188M, and L189M. In crystal form, the STC is locked in a single conformation *via* crystal lattice packing. In contrast, WT IN (1–278) was used to assemble the RSV octameric STC for structure determination by cryo-EM. These IN–DNA complexes are in-solution and hence free to adopt a wide variety of conformations. The cryo-EM structure of STC showed improved overall resolution (3.36 Å) and nearly 3 Å in the CIC region as compared to the crystal structure (3.86 Å). The CIC region of the STC obtained by cryo-EM was similar to CSC intasome (20) and the crystal structure of STC (15). Overall, the RMSD of C-α atoms in IN subunits was 0.805 to 1.1508 across 856 residues (Fig. S6) between the cryo-EM structure of RSV STC and its crystal structure (15). However, the NTD-CCD of the distal subunits remained flexible and poorly resolved in the cryo-EM structure of STC. In the crystal structure of the STC, the CCD of distal subunits was loosely associated with distal region of target DNA through nonspecific interactions. Similar to the cryo-EM structure of STC, there were no interactions identified beyond the 11th residue on target DNA from the viral–target DNA junction. Most of the interactions in the target DNA are limited to the CCD region of the proximal IN subunits which also contributes the active site (Figs. 2 and S2). Absence of IN interactions in outlying target DNA region possibly contributes to the flexibility observed in the distal NTD-CCD regions in the STC structure obtained by cryo-EM (Figs. S1*D* and S7).

We identified several potential IN–DNA interactions critical for CSC and STC intasome assemblies. Selected interactions (R244, Y246, and S124) were investigated for structure-function relationship studies using site-directed mutagenesis to determine their role in CSC and STC intasome assembly and catalytic activities. While this work was in progress, a study by Jozwik *et al*. (25) reported similar IN-viral and target DNA interactions in the MMTV STC intasome. RSV IN shares nearly 38% identity with MMTV IN; however, the CTD domain (222–268 aa) shows nearly 50% sequence identity (Fig. S8). R244 of the distal RSV IN subunit interacts with T3 at the 5′ terminal nontransferred strand in viral DNA, suggesting a critical binding point for distal IN subunits (Figs. 2 and S4). The proximal subunits interact with viral DNA at nucleotides 6-8 on nontransferred strand (Figs. 2, S2 and S4). We made substitution mutants R244A and R244E to establish the distal subunit DNA-binding properties. Both the R244A and R244E IN substitutions were unable to assemble the octameric CSC intasome (Fig. 3*A*) but could assemble the STC at a reduced capacity (Fig. 3*B*). It is probable that target DNA helped stabilize the STC intasome assembly by providing additional interaction sites. We previously showed that RSV IN (1–269) which produces a tetrameric CSC only is able to assemble octameric STC *in vitro* (4). The G7 nucleotide which interacts with R244 has been shown to be critical for concerted integration (26). It seems likely that R244 has multiple roles, that is, in binding to the viral DNA *via* proximal subunits as well in the CIC region assembly through interactions with the CTD of distal subunits (Figs. 2 and *S4*). Arginine at this position is conserved in a closely related MMTV IN (Fig. S8) and its substitution had similar effect on catalytic activities (14). The similar substitution in HIV-1 IN (K244A) makes the virus defective for replication (27).

The terminal three nucleotides from the 5′-end of the nontransferred end interacts with Y246 of the CTD from a distal subunit (Figs. 2, S2 and S4). IN Y246A assembled only the tetrameric CSC intasome with significantly reduced quantities of mature octameric CSC intasome, indicating its critical role in octameric CSC assembly (Fig. 3*A*). Whether this tetrameric CSC intasome containing two proximal dimers reflects the transient intermediate (4,5) in the concerted integration pathway is unclear and warrants further investigation. IN Y246A showed a moderate efficiency to assemble the STC intasome (Fig. 3*B*) and possessed decreased 3′-processing and concerted integration activity (Fig. 4). There appears to be no other apparent interactions of Y246 with target DNA in the STC intasome (Fig. S2). In addition, the possibility exists that 3′-OH processing occurs in the tetrameric intasome (Fig. 3*A*) because Y246 binds to T3 (distal subunit) and T5 (inner catalytic subunit of a proximal dimer) located on the 5′ end of the nontransferred DNA strand in the octameric intasome (Fig. S9) (20), which may be partially responsible for distortion of the DNA blunt ends necessary for 3′-OH processing (28). Y242 in MMTV IN is analogous to Y246 of RSV IN (Fig. S8) and predicted to have similar interactions with the viral DNA ends (25). The corresponding residue E246 in HIV-1 IN was shown to interact with viral DNA (29), and later studies determined that substitution at E246 to E246A/K affect 3′-processing and concerted integration (30, 31). The virions containing HIV-1 IN E246A/K substitutions were severely defective for replication (27). RSV IN W259 residue interacts with terminal nucleotide on nontransferred strand (Figs. 2, S2 and S4) and has also been shown to affect the CTD–CTD interaction of distal IN subunits. Substitutions of W259 to A/R/T in RSV IN abolished the 3′-processing and strand transfer activities (32). In summary, it seems possible that the disruption of Y246 (RSV IN) of the distal subunit interactions with the nontransferred viral DNA strand (Figs. 2 and S4) affects the recruitment of the distal subunits for octameric intasome assembly.

The CSC intasome assembly and its conversion to STC does not induce drastic conformational changes. Similar to RSV, earlier studies of PFV (13, 33) and Maedi-visna virus (16) intasomes, which are tetrameric and hexadecameric intasomes, respectively, also showed essentially no changes before and after target DNA binding. IN is well positioned to bind the target DNA in RSV octameric CSC intasome. The RMSD of C-α atoms in IN subunits was 0.668 to 1.132 across 917 residues (Fig. S10) between the cryo-EM structures of RSV CSC (20) and STC structures. There were minimal interactions between IN and six nucleotides immediately downstream of viral and target DNA junction. The crucial backbone-specific interactions are between S124 and eighth/ninth nucleotide downstream to the viral–target DNA junction site (Figs. S2–S4). A similar IN residue–nucleotide position interaction in the STC is conserved across multiple retrovirus species (34). The S124 residue in RSV IN and analogous S119 in HIV-1 IN was previously implicated in target site selection for integration and replication *in vivo* (24, 34, 35, 36). A number of substitutions of serine (124) to alanine, cysteine, aspartic acid, glutamic acid, asparagine, glutamine, lysine, or valine were investigated in oligonucleotide-based assays to determine effect on 3′-processing and nicking and joining reactions. The results suggested that both the charge and size of the substitutions affected the DNA binding and selection of integration sites. A small size and uncharged amino acid is conserved at this position in related retroviral INs, possibly due to limited amount of space available in the target DNA minor groove. S124 of RSV IN is analogous to S119 (HIV-1 IN), P125 (MMTV IN), and A188 in PFV IN (12). S124A substitution has no drastic effects on the CSC or STC intasome assembly (Fig. 5). As expected, S124A had 3′-processing and concerted integration activities similar to the WT IN (Fig. 4). These results are in full agreement with earlier studies including the fact that RSV virions with the S124A IN mutation replicates similarly to the WT RSV (24). In contrast, the RSV containing IN with the S124D substitution was unable to replicate in cell culture (35). An S124D substitution is unique that it separated the two biologically relevant enzymatic activities of IN. It allows the 3′-processing of the viral DNA substrates but blocks the strand transfer into host DNA. Our studies show that the S124D substitution had no effect on CSC intasome assembly (Fig. 5*A*), but STC intasome assembly was completely inhibited (Fig. 5*B*). Likewise, no strand transfer activities were detected (Fig. 4*B*) thus providing direct evidence that binding to the target DNA is affected. In summary, our studies of S124 provide evidence for its role in RSV CSC and STC intasome assemblies and structural rationale into its effect on catalysis and viral replication.

Collectively, our data advance the understanding of RSV intasome structure and function and identified molecular determinants involved in intasome assembly. We provided additional evidence to show the role of CTD in viral DNA binding and recruitment of distal subunits to form mature octameric CSC intasomes. Whether the tetrameric to octameric CSC transition occurs in virus infected cells remains to be determined. Currently, such studies are limited by technical feasibilities of obtaining sufficient quantities of preintegration complexes for structural studies. Furthermore, viral RNA and complementary DNA formed after reverse transcription is bound with IN along with close association with RT among other viral and cellular components (37). The terminal 200 to 250 bp of HIV-1 U3 and U5 LTR ends of HIV-1 were protected by bound proteins from nuclease digestion in footprinting assays, dependent on IN catalytic activity (38). Advancements in single molecule imaging methods and determination of preintegration complex structures during various stages of viral infection and replication would be instrumental to address these important questions.

## Experimental procedures

### RSV IN expression and purification

RSV IN constructs (WT, C-terminal tail truncations, and single-point mutants) were expressed in *Escherichia coli* BL21 (DE3) pLysS and purified to near-homogeneity (3, 4, 5). The WT Prague A IN subunit is 286 aa in length, designated 1 to 286. Purified IN were concentrated to 20 to 30 mg/ml using Amicon Ultra-15 (30K MWCO) centrifugal filters. IN mutant expression constructs (R244A, R244E, Y246A, S124A, and S124E were produced in full length RSV IN (1–286) by site-directed mutagenesis. The NTD-CCD and CCD-CTD linker region of RSV IN (1–286) were modified to mimic the corresponding regions from PFV IN (13) either individually or together. The modified construct having the 26 aa NTD-CCD linker and the 45 aa CCD-CTD linker were named N-C 26 and C-C 45, respectively. The IN construct possessing both linkers was termed NC/CC (26/45). The DNA sequence of all IN constructs was confirmed by sequencing. The protein concentrations were expressed as monomeric subunits.

### Assembly and purification of the RSV STC intasome

The RSV octameric STC intasome was assembled with IN 1 to 278 and the STC DNA substrate (4, 15). IN (1–278) is more efficient in producing CSC and STC intasomes than IN (1–286) (4, 20). The STC substrate was prepared by annealing three oligonucleotides in equimolar ratios (42 nt 5′-GAGTATTGCATAAGACAACAGTGCACGAAAGAAGAAGACACT-3′, 22 nt 5′-AATGTTGTCTTATGCAATACTC-3′, and 16 nt target 5′-AGTGTCTTCTTCTTTC-3’). The IN and STC substrate (35 μM and 10 μM, respectively) were mixed in 20 mM N-(2-hydroxyethyl)piperazine-N′-(2-ethanesulfonic acid) (HEPES), 1 M NaCl, 20% glycerol, 1 mM tris(2-carboxyethyl phosphine) (TCEP) pH 7.5 and dialyzed in a Slide-A-Lyzer G2 cassette (3.5 K cutoff) overnight against 20 mM HEPES, 0.125 M NaCl, 20% glycerol, and 1 mM TCEP), pH 7.5. Precipitated intasomes were solubilized by dialysis against a higher salt buffer containing 20 mM HEPES, 0.75 M NaCl, 20% glycerol, and 1 mM TCEP, pH 7.5, for 1 h at room temperature. The octameric RSV STC intasomes were purified by SEC using Superdex 200 Increase column (10/300) (Cytiva Life Sciences) (Fig. 1*C*). The SEC running buffer was 20 mM HEPES, pH 7.5, 650 mM NaCl, and 1 mM TCEP. SEC-purified fractions were used immediately for vitrification. Rechromatography of pooled STC fractions on Superdex 200 established that the octameric RSV STC intasomes were stable on ice even after overnight storage at 4 ºC.

### Concerted integration and 3′-OH processing assays

The concerted integration assays were performed using 3′-OH–recessed oligonucleotide viral DNA substrates with RSV IN as previously described (3, 4). Double stranded 3′-OH–recessed substrates containing RSV gain-of-function (G) U3 and WT U3 LTR sequences were 18 nucleotides in length and synthesized by Integrated DNA Technologies. The DNA substrates were recessed by two nucleotides on the catalytic strand and designated with an R. The identified length of the oligonucleotide denotes the noncatalytic strand. The sequences were as follows: GU3 18R (5′-ATTGCATAAGAC**A**ACA-3′ and 5′-AATGT**T**GTCTTATGCAAT-3′). The bold underlined nucleotide on the catalytic strand was different between the GU3 and WT U3 sequence. The concentrations of IN and the viral LTR substrate in a typical assay were 2 and 1 μM, respectively. The strand transfer products were separated on a 1.3% agarose gel, stained with SYBR Gold (Invitrogen), and analyzed by a Typhoon 9500 Laser Scanner (GE HealthCare Life Sciences).

The 3′-OH processing of ^32^P-labeled blunt-ended viral 4.6 kb DNA at 37 °C was previously described (32, 39). Concentrations of purified IN and DNA in the assay mixture was 20 nM and 0.5 nM, respectively.

### Assembly of the RSV CSC

To determine the effect of IN mutations, the RSV CSC was assembled with IN and GU3 18R in the presence of MK-2048 (3). The assembly buffer was 20 mM HEPES, pH 7.5, 100 mM NaCl, 100 mM ammonium sulfate, 1 M nondetergent sulfobetaines-201, 10% dimethyl sulfoxide, 10% glycerol, 1 mM TCEP. IN (as monomers), 3′-OH–recessed DNA GU3 18R and MK-2048 concentrations were 45 μM, 15 μM, and 125 μM, respectively. MK-2048 was generously provided by Merck & Co. The samples were generally incubated at 18 °C for 18 h, unless indicated otherwise. The octameric RSV CSC intasomes were analyzed by SEC using Superdex 200 Increase (10/300). The SEC running buffer was 20 mM HEPES, pH 7.5, 200 mM NaCl, 100 mM ammonium sulfate, and 1 mM TCEP. MK-2048 was omitted from the running buffer. Chromatography was performed at 4 °C and UV absorption monitored at 280 nm.

### Cryo-EM sample preparation of the RSV STC and imaging

The cryo-EM samples derived from the peak SEC fractions (∼0.4 μM) were used promptly for vitrification. The samples were prepared on quantifoil holey carbon grids (R2/2 300 mesh copper), which were plasma cleaned for 1 min using a Gatan Solarus 950 (Gatan) and plunge frozen using a Vitrobot Mark IV (Thermo Fisher Scientific). The Vitrobot sample chamber was set to 4 °C and 100% humidity. Three microliters of SEC-purified RSV STC was applied to the plasma-cleaned quantifoil grids and allowed to incubate for 20 s. Samples were then blotted for 2 s at a blot force of −1 and plunge frozen into liquid ethane. Vitrified grids were imaged using a Cs-corrected Thermo Fisher Titan Krios G3 electron microscope (Thermo Fisher Scientific) operating at an accelerating voltage of 300 kV equipped with a Falcon 4 detector (Thermo Fisher Scientific). Data acquisition was automated using EPU software (Thermo Fisher Scientific, https://www.thermofisher.com/us/en/home/electron-microscopy/products/software-em-3d-vis/epu-software.html) at a magnification of 590,00× which corresponds to a pixel size of 1.16 Å. Movies were recorded for 13.29 s with 50 frames with a dose rate of 1.0 electrons per Å^2^ per frame (total dose of 50 electrons per Å^2^). The defocus was varied between −1 to −2.5 μm. A total of 3297 movies were recorded including 248 movies at a 20-degree stage tilt. The data collection parameters are indicated in Table S1.

### Cryo-EM data processing

The movies were corrected for beam-induced movement using MotionCorr2 (40). Further data analysis was done in cryoSPARC 3.0 (Fig. S1) (21). The contrast transfer function (CTF) was determined using Patch-based CTF estimation. The micrographs with estimated CTF fit resolution in range of 2.4 to 6.0 Å were selected for further analysis. Initially, the blob picker was used to pick the particles of diameter 120 to 200 Å from 500 micrographs. The particles were extracted using a box size of 324 pixel and reference-free 2D classification performed. The selected 2D classes showing different conformations were used as a template to pick particles from all micrographs. The 867, 209 particles were subjected to 2D classification in 100 classes. After removing the junk particles, selected 2D class averages containing 214, 690 particles were used for Ab-initio 3D reconstruction. The 3D reconstructions were refined by homogeneous refinement without imposing symmetry. The class with clear feature showed a global resolution map of 3.98 Å at Fourier shell correlation of 0.143. The nonuniform refinement of this class using C2 symmetry resulted in a 3.36 Å global resolution map and was used for model building and refinement. Heat map of angular distribution of refined particles used in reconstruction is shown in (Fig. S1*G*). Directional 3DFSC curves were calculated using the wrapper program within the cryoSPARC (41). The local resolution in the CIC region was determined in cryoSPARC and displayed in Chimera (42) (Fig. S1*H*). The local resolution map demonstrated ∼2.8 to 4.0 Å resolution in the core region.

### Preparation of the atomic model, refinement, and validation

Our previously reported RSV STC crystal structure at 3.86 Å resolution (PDB ID 5EJK) was used as the preliminary model to dock into the EM map as a rigid body and manually modified/rebuilt using Coot (https://www2.mrc-lmb.cam.ac.uk/personal/pemsley/coot/) (43). The preliminary model was refined using PHENIX (https://phenix-online.org) (44, 45) against the cryo-EM density and a standard set of geometry/stereochemistry restraints.

## Data availability

The cryo-EM maps were deposited with the Electron Microscopy Data Bank (accession code EMDB-27823) and the refined model with the Protein Data Bank (8E14). All materials used in the manuscript are available upon request.

## Supporting information

This article contains Supporting Information (46) (Figs. S1–S10 and Table S1).

## Conflict of interest

The authors declare that they have no conflicts of interest with the contents of this article.
