# Supplementary material for: Molecular determinants for Rous sarcoma virus intasome assemblies involved in retroviral integration
Source: J Biol Chem. 2023 Apr 20;299(6):104730. doi: 10.1016/j.jbc.2023.104730 (PMC10209032; doi:10.1016/j.jbc.2023.104730)
Supplement: Supporting information [file mmc1.pdf]

Supporting information for

Molecular determinants for Rous sarcoma virus intasome assemblies involved in retroviral integration

Sibes Bera[1], Ke Shi[2], Hideki Aihara[2], Duane P. Grandgenett[1], and Krishan K. Pandey[1,3]

[1] Department of Molecular Microbiology and Immunology, School of Medicine, Saint Louis University, St. Louis, MO 63104 USA

[2] Department of Biochemistry, Molecular Biology and Biophysics, University of Minnesota, Minneapolis, MN 55455 USA

[3] Corresponding author: krishan.pandey@health.slu.edu

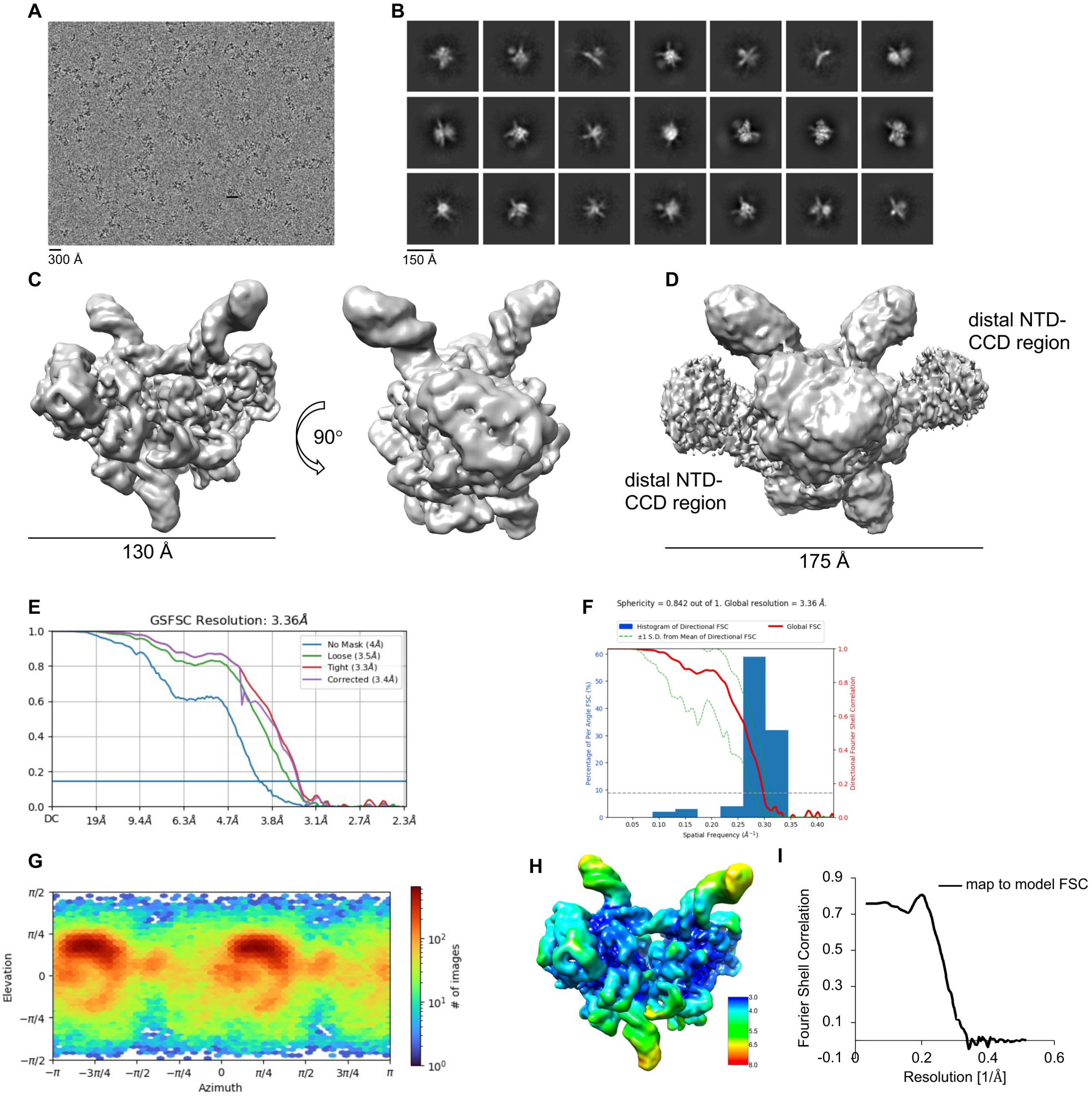

Fig S1. Cryo-EM data analysis workflow. A. Representative cryo-EM micrograph. **B.** Representative reference free 2-D class averages of the particles after template picking. **C.** Non-uniform refinement of the 3D map using C2 symmetry. **D.** Same map as in C shown with lower threshold to show flexible NTD-CCD regions of distal dimer subunits. **E.** Gold standard Fourier shell correlation curves (FSC) for the half-maps. **F.** Global resolution FSC curve overlaid onto a histogram of direction resolution value. **G.** Particle viewing directional distribution plot of the RSV STC. **H.** Reconstruction of STC map colored by local resolution. **I.** Map to model FSC plot. Detailed description of data analysis is provided in Experimental Procedures.

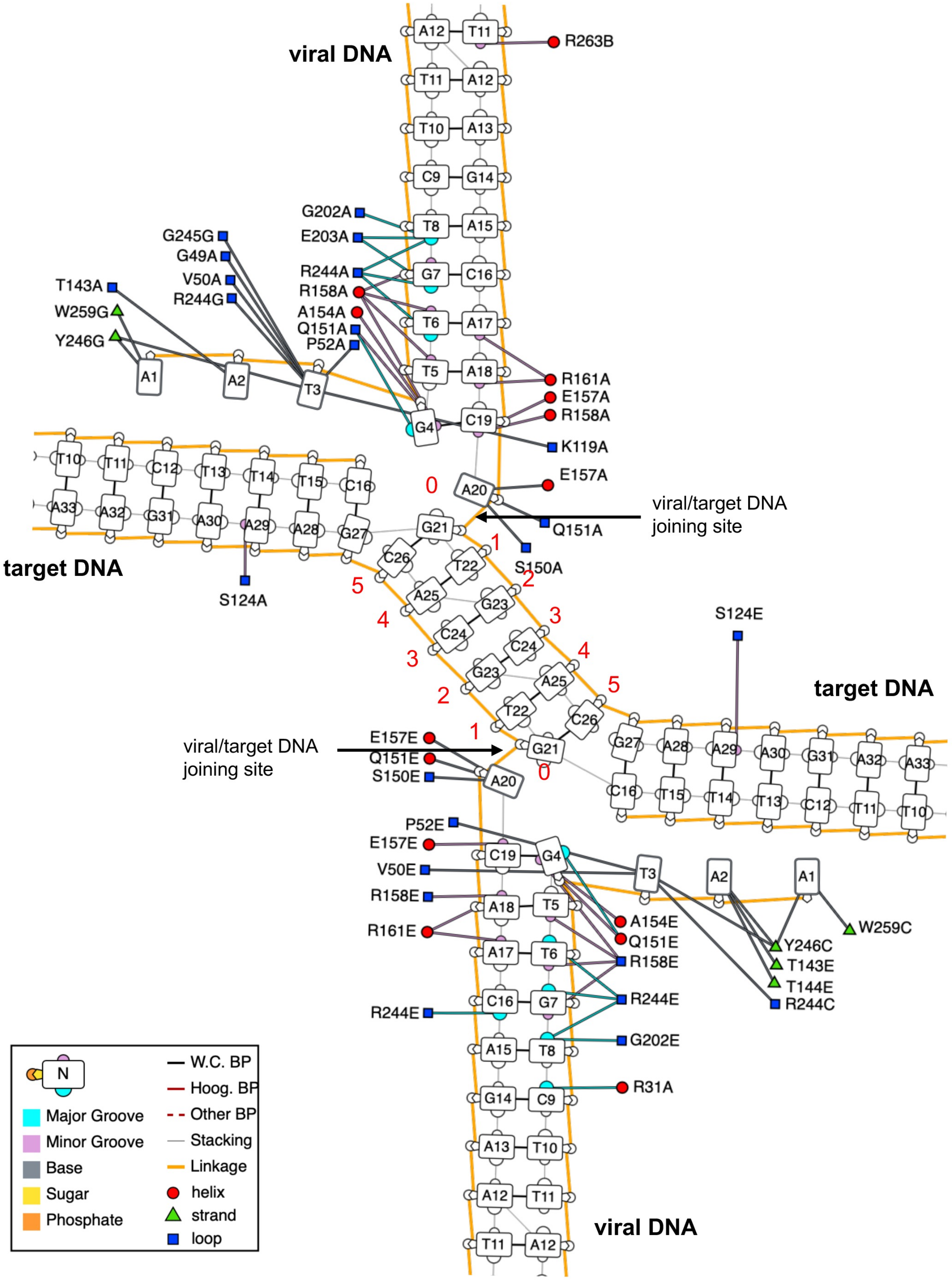

Fig S2. Potential nucleotide base-specific interactions between IN and DNA in the RSV STC. Analyses were performed using DNAproDB to determine nucleotide base specific interactions including major and minor groove. Most of the IN interactions are with viral DNA and minimal interaction with target DNA occurs. Protein residues are labeled with their one letter codes, respective residue number and the protein chain identifier (*e.g.*, R244E indicate R244 of chain E). The protein chains follow the same designation as in Fig. 1E. The 6 bp host site duplication sequence is labeled 0-5 in red. The site joining the viral DNA to target DNA is marked. Schematic shows the potential interactions between IN subunits and nucleotide bases, major and minor grooves. W.C. BP – Watson–Crick bp; Hoog. BP – Hoogsteen bp; Other BP – other bp.

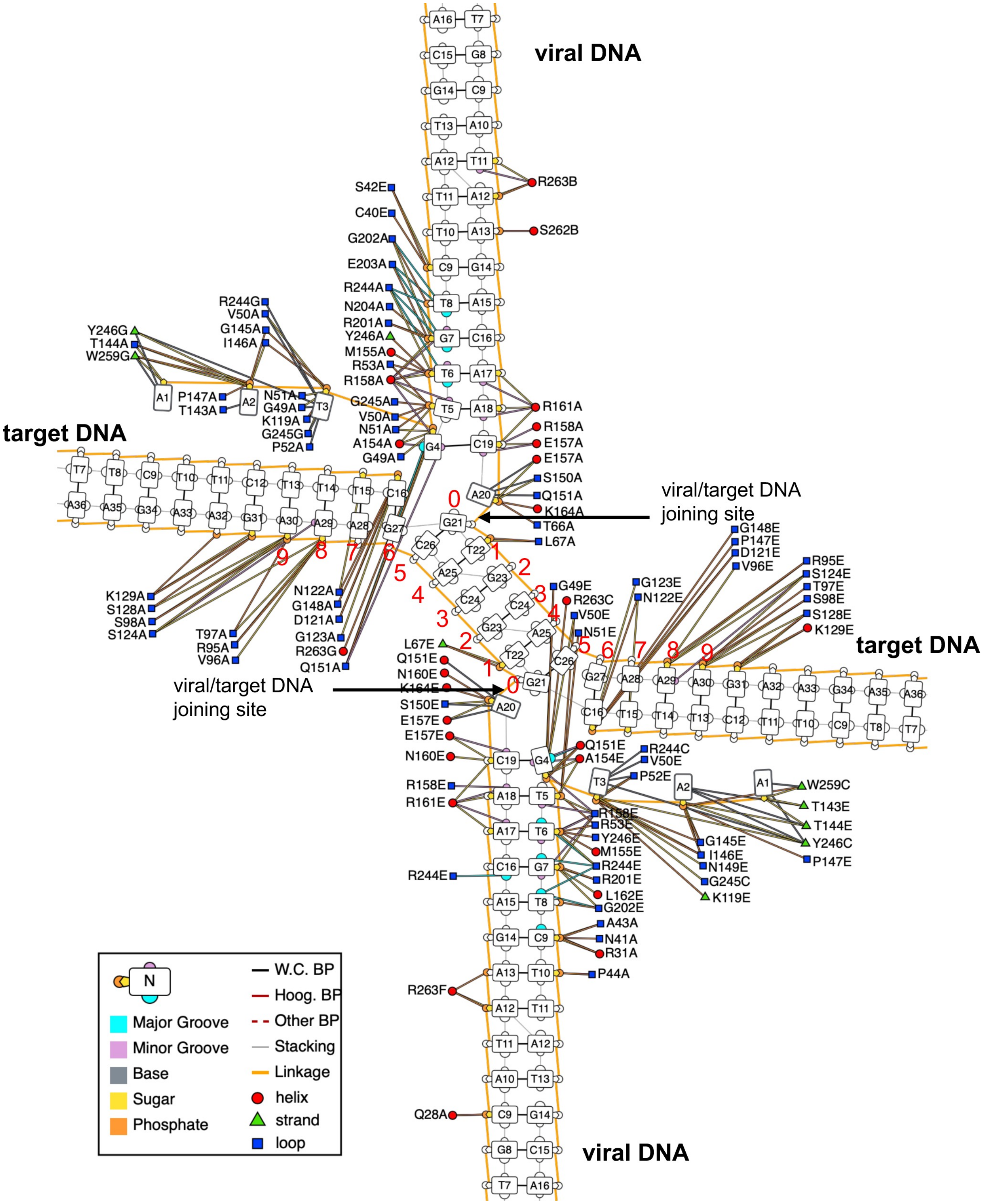

Fig S3. Potential backbone interactions between IN and DNA in the RSV STC. The analyses were performed using DNAproDB. There were minimal interaction in the six bp duplication region at the viral-target DNA junction. The majority of the IN interactions with target DNA are in region beyond the 6 bp duplication region. Protein residues are labeled with their one letter codes, respective residue number, and the protein chain identifier (*e.g.*, R244E indicate R244 of chain E). The protein chains follow similar designation as in Fig. 1E. The 6 bp host site duplication sequence is labeled 0-5. The site joining the viral DNA to target DNA is marked. Schematic shows the interactions between IN protomers and nucleotide bases, major and minor grooves. W.C. BP – Watson–Crick bp; Hoog. BP – Hoogsteen bp; Other BP – other bp.

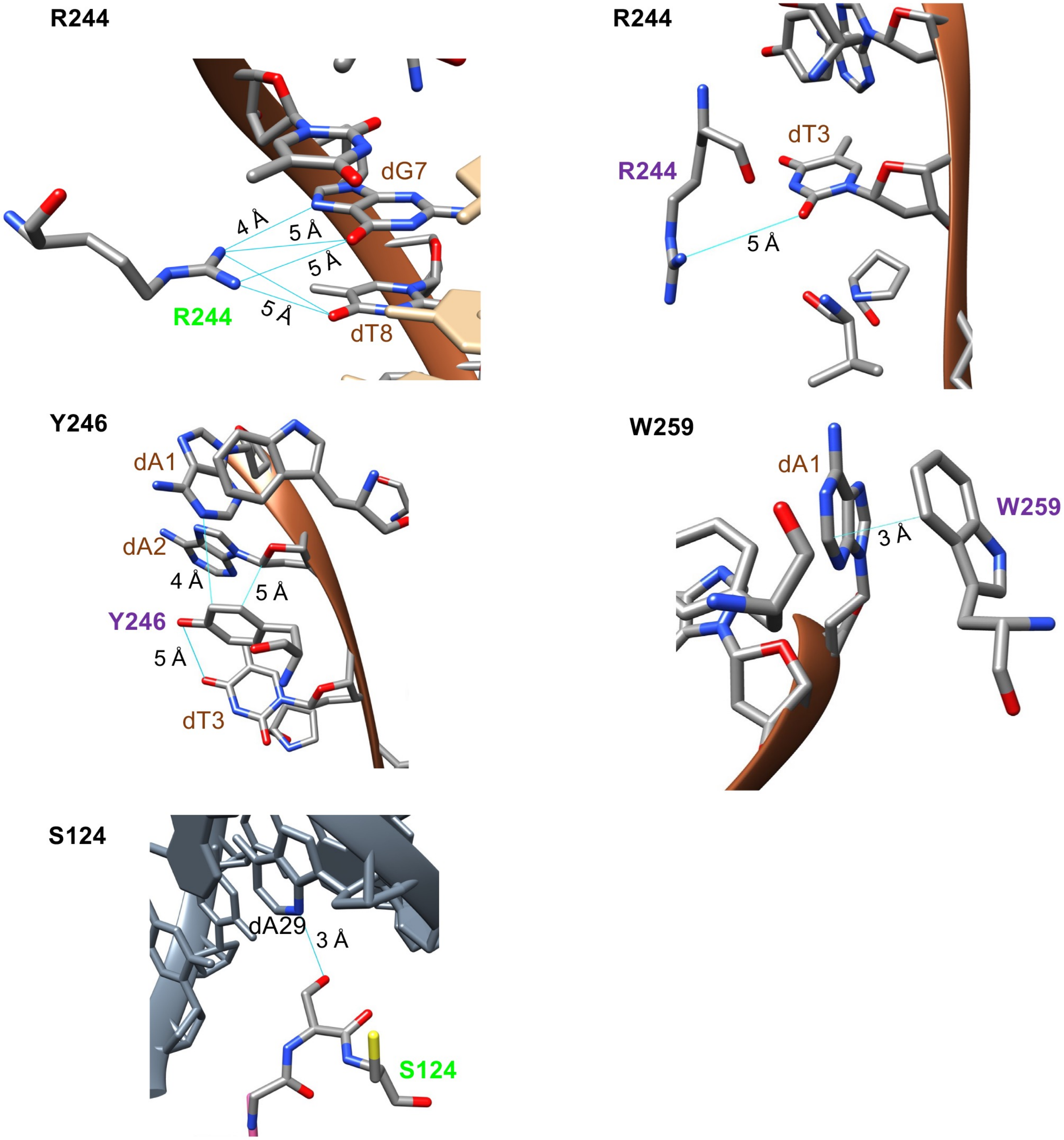

Fig. S4. Detailed view of select potential IN-DNA interactions in RSV STC. The IN residues from proximal subunits are in green while the distal IN residues are shown in purple. The specific nucleotides on viral and target DNA interacting with IN residues are labeled and numbered starting from 5'-end of the oligonucleotides. The distance between IN and nucleotides for each interactions is marked.

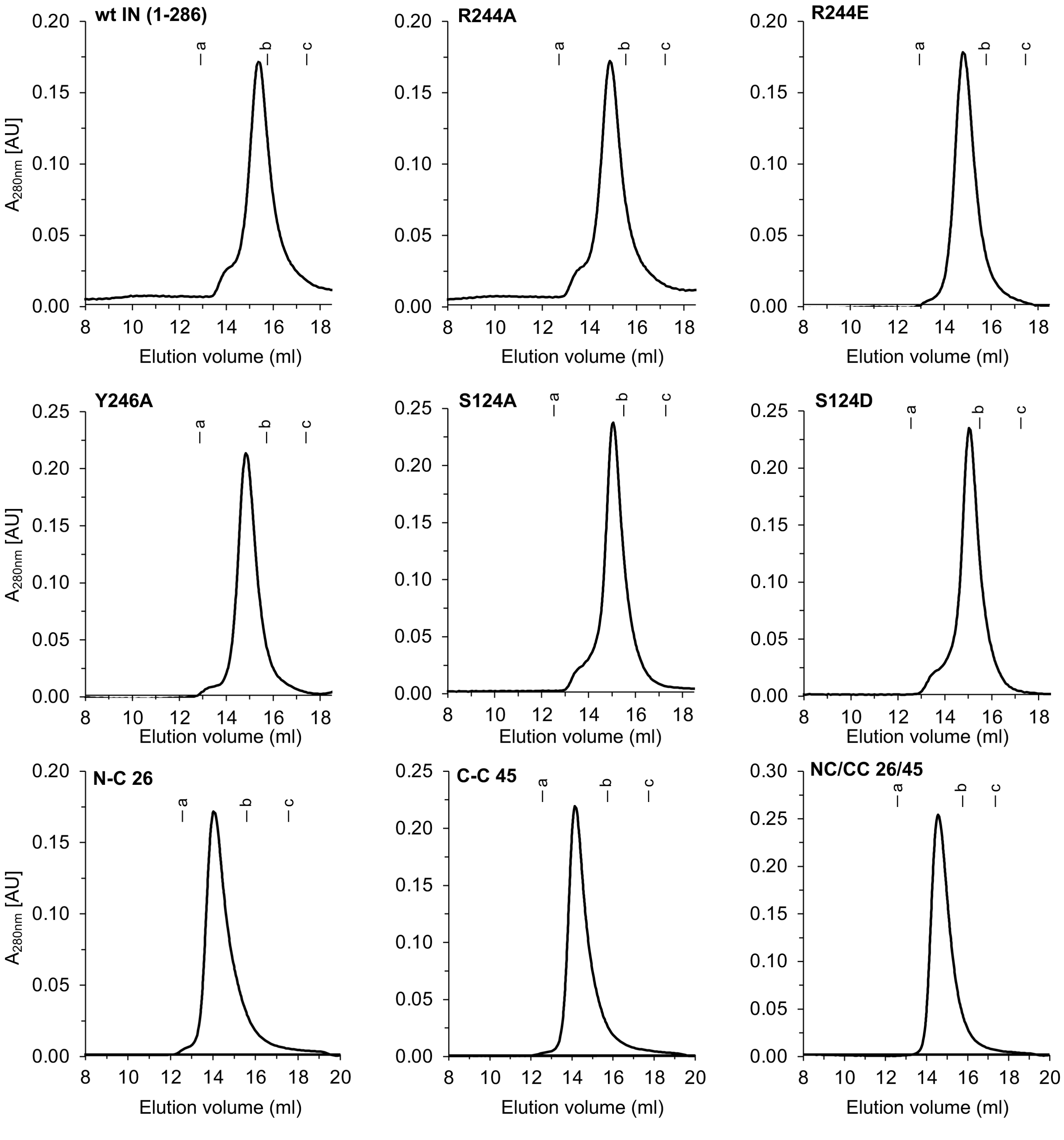

Fig S5. **Oligomeric forms of RSV IN mutants.** wt RSV IN (1-286) and its indicated variants containing missense substitutions and alterations in the inter-domain linker length were analyzed by SEC using Superdex 200 Increase column (10 x 300 mm). All of the IN proteins at 45 μM concentrations were incubated in the CSC intasome assembly buffer overnight at 4°C before SEC analysis. IN eluted predominantly as dimeric species. The molecular weight standards were run in parallel with each analysis and their elution positions are marked on top of each profile with a, b, and c which corresponds to 158 kDa, 44 kDa, and 17 kDa respectively. The slight variation in relative elution positions among different SEC run was due to use of a new column for few samples.

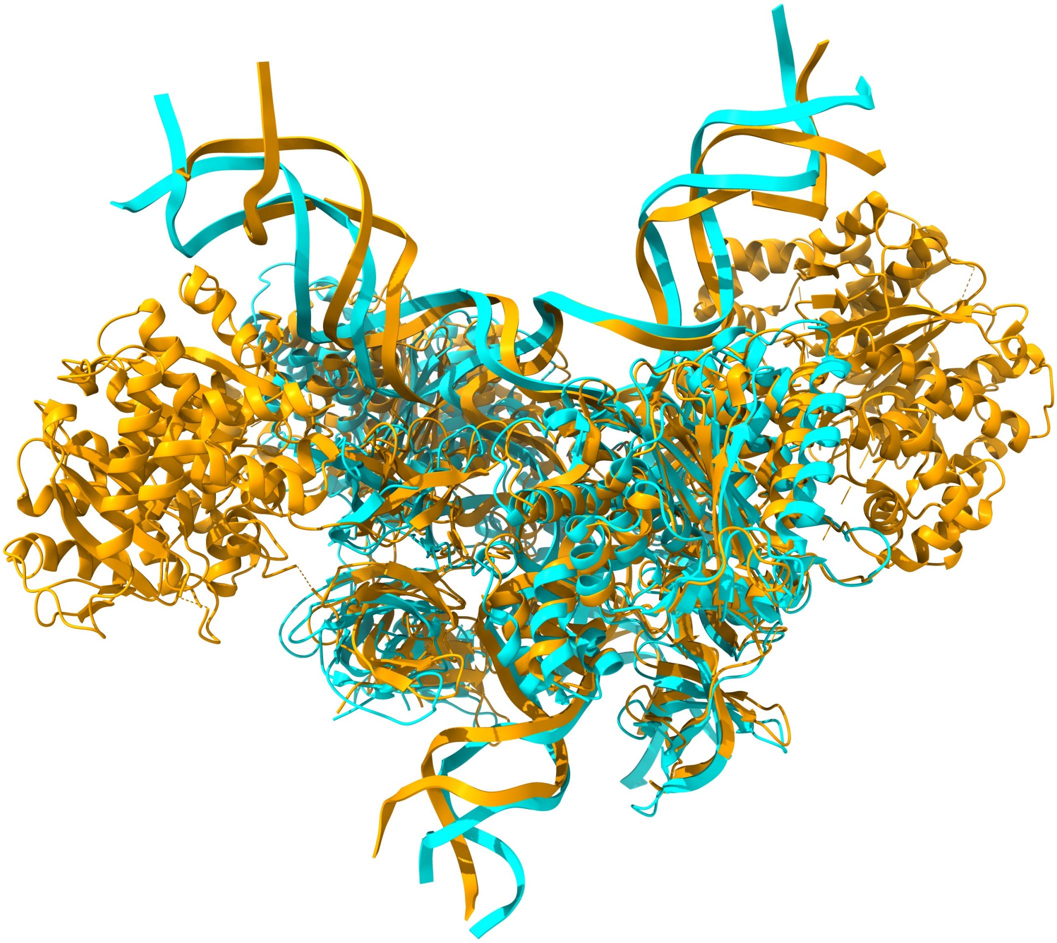

Fig. S6. Alignment of the RSV STC structure by cryo-EM (in cyan) and x-ray crystallography (PDB 5EJK) in orange color.

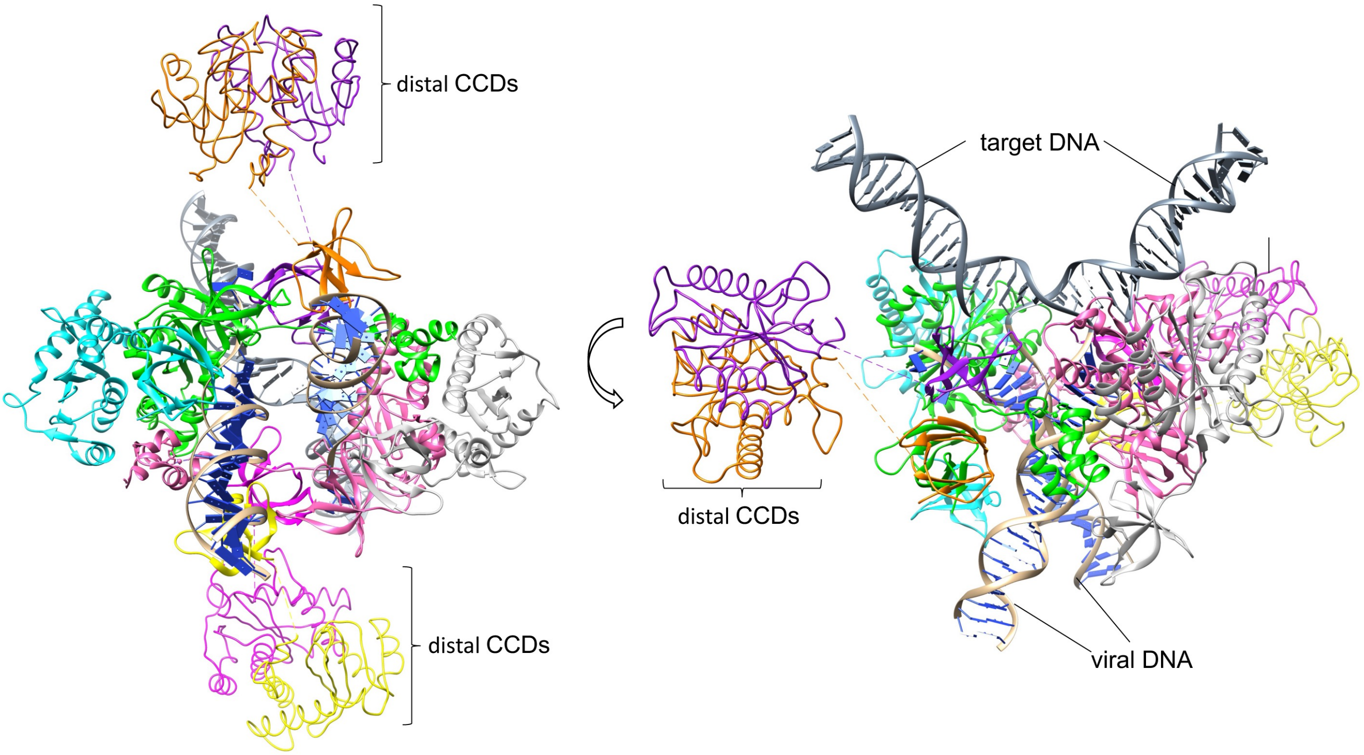

Fig. S7. Model of the whole RSV intasome. The backbone of distal CCD subunits were modeled based on their electron densities. The individual subunits follow same coloring scheme as in Fig. 1E.

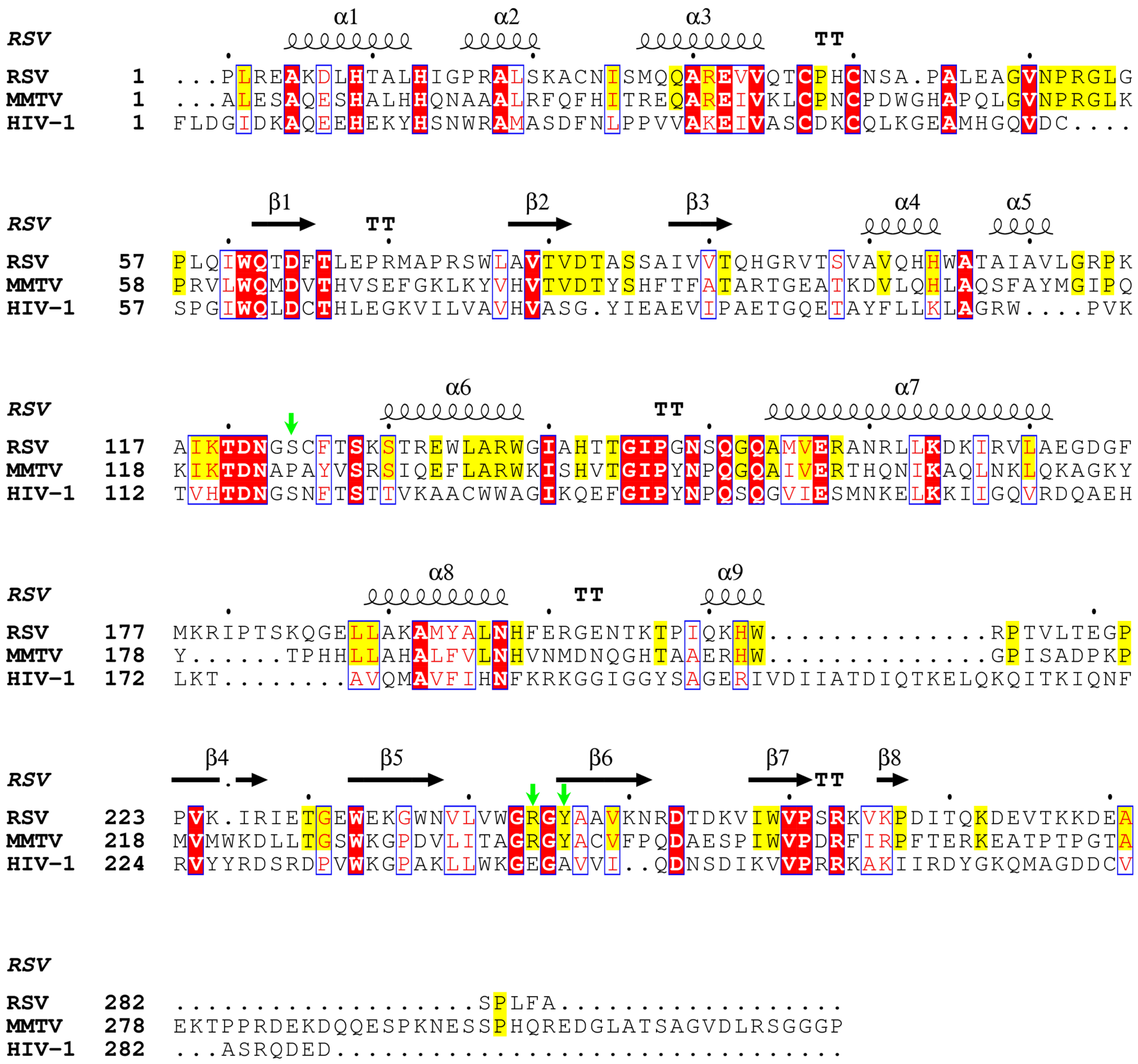

Fig. S8. Amino-acid sequence alignment of RSV, MMTV and HIV-1 IN. The secondary structure elements for RSV IN are derived from the structure shown in Fig. 1E. The residues numbering at the top of is for RSV IN. The residues conserved across all three IN are shown in bold and red background. The residues conserved between RSV and MMTV IN are shown in yellow background. The RSV IN residues marked with green arrows were investigated in this study for their role in CSC and STC intasome assembly and catalytic activities. This figure was prepared partially using ESPript (46).

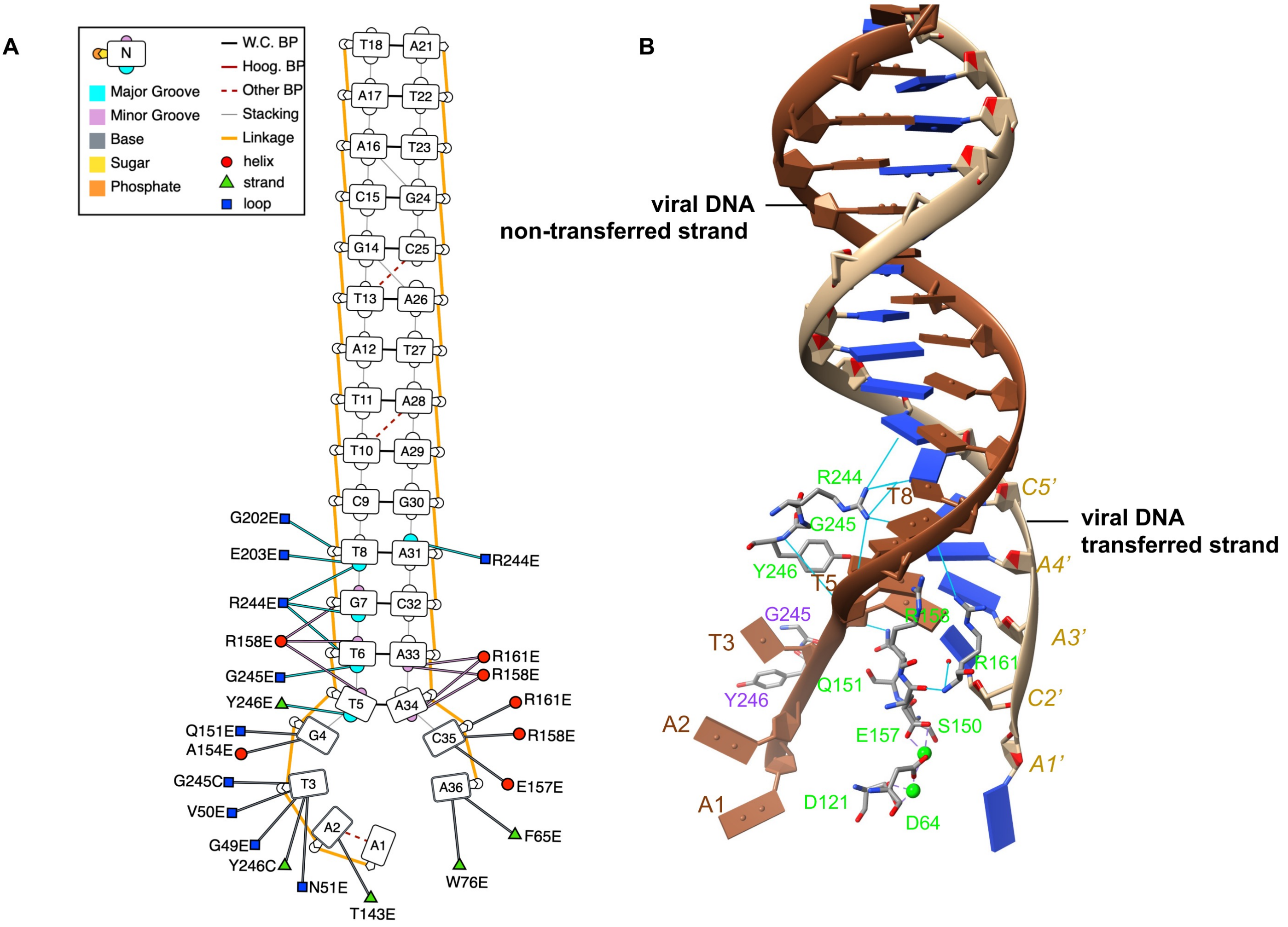

Fig S9. Critical IN-viral DNA contacts in RSV octameric CSC intasome. A. Potential nucleotide base-specific interactions including major and minor groove between IN and DNA in the RSV octameric CSC intasome. Analyses were performed using DNAproDB on octameric CSC intasome (PDB 7JN3). Protein residues are labeled with their one letter codes, respective residue number and the protein chain identifier (*e.g.*, R244E indicate R244 of chain E). The protein chains follow the same designation as in Fig. 1E. Schematic shows the potential interactions between IN subunits and nucleotide bases, major and minor grooves. W.C. BP – Watson–Crick bp; Hoog. BP – Hoogsteen bp; Other BP – other bp. **B.** Visualization of select IN-viral DNA interactions in CSC intasome. The select IN residues of proximal and distal subunits interacting with viral DNA are colored by their respective protein chain as in Fig. 1E.

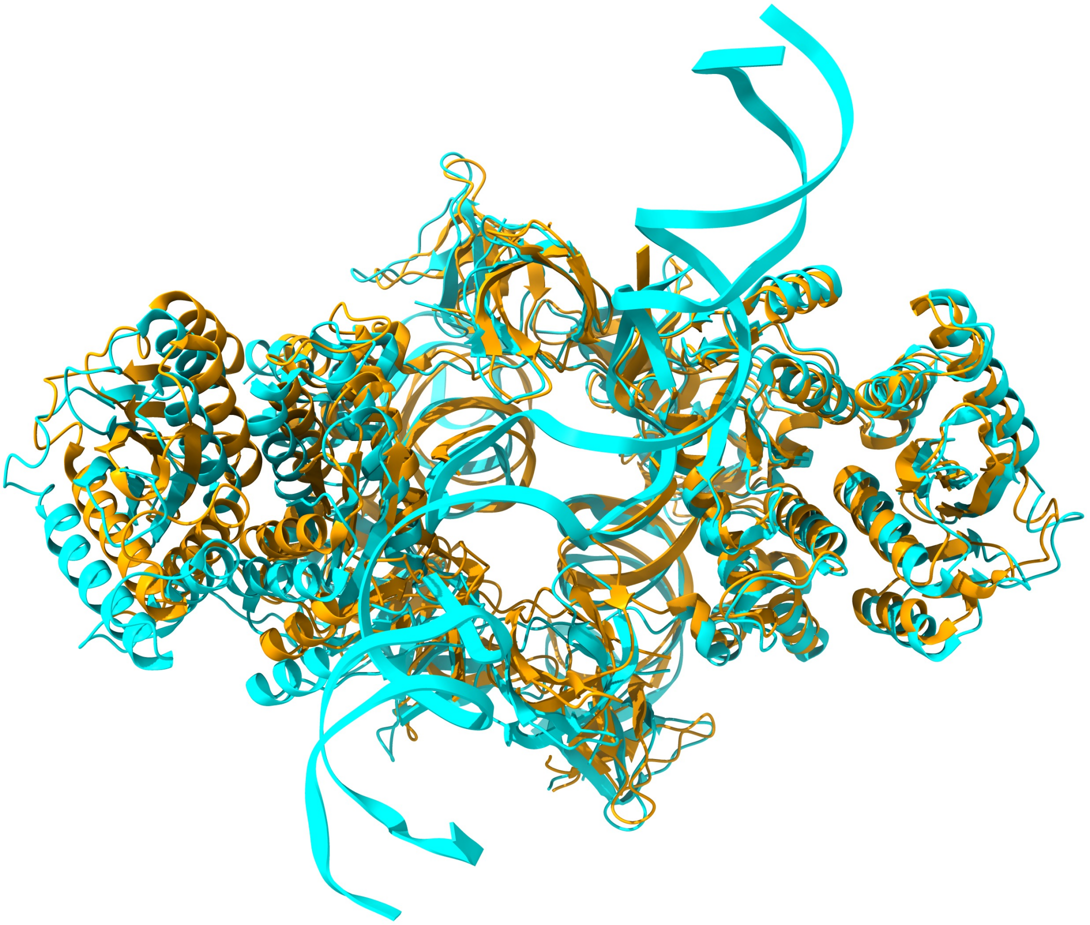

Fig. S10. Comparison of the CIC region in RSV CSC intasome (PDB 7JN3, shown in orange) and STC (in cyan) determined by cryo-EM.

Supporting Table 1. cryo-EM data collection, refinement and validation statistics.

	**RSV STC (EMDB-27823, PDB 8E14)**
**Data collection and processing**	
Microscope	Titan Krios
Detector	Falcon 4
Magnification	59,000 X
Voltage (kV)	300
Electron exposure (e^-/Å^2)	50
Defocus range (μm)	0.8-2.5
Pixel size (Å)	1.16
Total movies acquired/used	3297
Symmetry imposed	C2
Initial particle images (no.)	8,67,209
Final particle images (no.)	141,428
Map resolution (Å)	3.36
FSC threshold	0.143
Map resolution range (Å)	2.8-4.0
**Refinement**	
Initial model used (PDB code)	5EJK
Model resolution (Å)	3.36
FSC threshold	0.142
Map sharpening *B* factor (Å^2)	100
Map CC	0.84
Model composition	
Non-hydrogen atoms	12442
Protein residues	1161
DNA residues	160
Metal ions (Zn^{++}, Mg^{++})	2
Ligand (MK-2048)	2
B factors (Å^2)	
Protein	198.82
DNA	4214.53
R.m.s. deviations	
Bond lengths (Å)	0.004
Bond angles (°)	0.720
Validation	
MolProbity score	2.42
Clashscore	16.23
Poor rotamers (%)	0.31
Ramachandran plot	
Favored (%)	82.27
Allowed (%)	17.73
Disallowed (%)	0
